# Resource Allocation in a National Dental Service Using Program Budgeting Marginal Analysis

**DOI:** 10.1177/23800844211056241

**Published:** 2021-11-29

**Authors:** C.R. Vernazza, K. Carr, R.D. Holmes, J. Wildman, J. Gray, C. Exley, R.A. Smith, C. Donaldson

**Affiliations:** 1 School of Dental Sciences, Newcastle University, Newcastle upon Tyne, UK; 2 Population Health Sciences Institute, Newcastle University, Newcastle upon Tyne, UK; 3 Nursing, Midwifery & Health, Northumbria University, Newcastle-upon-Tyne, UK; 4 ScHARR, University of Sheffield, Regent Court, Sheffield, UK; 5 Yunus Centre for Social Business and Health, Glasgow Caledonian University, Glasgow, UK

**Keywords:** health economics, priority setting, preference elicitation, oral health, dental care, health policy

## Abstract

**Introduction:**

In any health system, choices must be made about the allocation of resources (budget), which are often scarce. Economics has defined frameworks to aid resource allocation, and program budgeting marginal analysis (PBMA) is one such framework. In principle, patient and public values can be incorporated into these frameworks, using techniques such as willingness to pay (WTP). However, this has not been done before, and few formal resource allocation processes have been undertaken in dentistry. This study aimed to undertake a PBMA with embedded WTP values in a national dental setting.

**Methods:**

The PBMA process was undertaken by a panel of participant-researchers representing commissioners, dentists, dental public health staff, and academics. The panel reviewed current allocations and generated a set of weighted criteria to evaluate services against. Services to be considered for removal and investment were determined by the panel and wider discussion and then scored against the criteria. Values from a nationally representative WTP survey of the public contributed to the scores for interventions. Final decisions on removal and investment were taken after panel discussion using individual anonymous electronic voting.

**Results:**

The PBMA process resulted in recommendations to invest in new program components to improve access to general dentists, care home dentistry, and extra support for dental public health input into local government decisions. Disinvestments were recommended in orthodontics and to remove routine scaling and polishing of teeth.

**Discussion:**

The PBMA process was successful in raising awareness of resource allocation issues. Implementation of findings will depend on the ability of decision makers to find ways of operationalizing the decisions. The process illustrates practical aspects of the process that future dental PBMAs could learn from.

**Knowledge Transfer Statement:**

This study illustrates a framework for resource allocation in dental health services and will aid decision makers in implementing their own resource allocation systems.

## Introduction

Within health care systems, there is insufficient resource to provide all possible services to the population. Therefore, decisions need to be made about how to allocate scarce resources. Considerations when making these decisions are likely to include allocative efficiency, which would mean each additional pound spent across services would yield the same health gain. However, this is difficult to define, given challenges in measuring and valuing health and in predicting health outcomes from health care used (Donaldson and Shackley 1997). In reality, health care systems also have multiple objectives, sometimes competing, with decisions to allocate resources to satisfy these multiple objectives often requiring normative judgments about trade-offs.

Instead of confronting these challenges, resource allocation in health often tends to be based on perpetuating historical allocations or allocating resources to those who are best at making a case (the “decibel” approach) (Mitton and Donaldson 2004). While economic evaluation techniques of cost-effectiveness, cost-utility, and cost-benefit analysis can provide useful information to feed into decisions, there are limitations to these approaches. Even if an intervention is of value (e.g., based on an incremental cost-effectiveness ratio [ICER] or cost-benefit ratio), in a system with fixed budgets, these methods do not aid decision makers with overall resource allocation. The introduction of a new intervention (even if it is cost-effective) will, by definition with fixed budgets, lead to some other service losing funding. However, in economic evaluation, this trade-off is implicit. One example of this is program budgeting and marginal analysis (PBMA) (Mitton et al. 2014).

PBMA has been widely used in health care, although mostly at a regional or local level (Kapiriri and Razavi 2017). While still reliant on some subjective judgments, PBMA allows a more evidence-based and transparent decision-making process to be undertaken (Hall, Mitton, et al. 2018). It has been suggested that indications of the success of PBMA are 4-fold: greater understanding of the resource allocation problem, complete or partial implementation of the PBMA recommendations, actual reallocation of resources, and adoption of PBMA for future use (Tsourapas and Frew 2011). However, there has been a recognition that, for PBMA projects to be successful in terms of embedding in organizational decision making, there is a need for practical guidance on conducting them (Hall, Williams, et al. 2018).

The importance of involving patients and the public in resource allocation decisions is well established and has typically been done through the inclusion of patient or public representatives on panels (Mitton et al. 2009). However, concerns have been raised about how representative a small number of volunteers can truly be. Decision makers are therefore cautious about the feasibility and utility of incorporating public views (Daniels et al. 2018). An alternative approach is to undertake larger-scale surveys of patient/public views using representative samples. Health economics has several methodologies for eliciting such preferences, one of which is to establish maximum societal willingness to pay (WTP) as a measure of the community’s strength of preference for different services (Donaldson and Shackley 1997). However, to the authors’ knowledge, WTP has not yet been incorporated into a PBMA for health care resource allocation.

In the United Kingdom, most health care is provided under the publicly funded National Health Service (NHS), which, in the main, is free at the point of delivery. Dentistry, however, is treated differently, with patients paying a significant copayment (with exceptions for specific groups). In England, the NHS directly engages in a contract with independent dental providers (Steele 2009). Previous work has shown that there is little explicit resource allocation decision making in dentistry in England (Vernazza et al. 2019). To the authors’ knowledge, only 1 PBMA has been undertaken in dentistry and was limited to 1 region of England (Holmes et al. 2018). While this was judged a success in terms of increased awareness, recommendations for reallocations were not enacted due to a change in the structure of the NHS.

This study, therefore, aimed to undertake a PBMA process for NHS dentistry in England at a national level, incorporating public views through a WTP survey. The article aims to describe the process as a case study in such a way as to show learning from the process and provide practical guidance for future dental PBMA processes.

## Methods

The PBMA was the main element of the RAINDROP (Resource Allocation in NHS Dentistry: Recognition of Societal Preferences) project. The detailed methods for the overall project are described in a protocol paper (Vernazza et al. 2018). The project was reviewed by Newcastle University Ethics Committee (reference Nos. 00873/2015 and 7065/2016). This article focuses on the conduct and results of the PBMA process, which followed that described by Peacock et al. (2006). A participatory action research approach (Whyte 1991) was adopted in which the members of the PBMA panel were also considered part of the research team, shaping the project as it progressed. The PBMA followed the 7 steps as described by Peacock et al. (2006):

1. Determine the aim and scope of the priority setting exercise.
2. Compile a “program budget,” describing current resources used and activity information for program components.
3. Form a “marginal analysis” advisory panel.
4. Determine relevant decision-making criteria (e.g., maximizing benefits, improving access and equity), with reference to specified objectives of the health system and the community, and weight these for relative importance.
5. Among program components, identify options for a) service growth, b) resource release from operational efficiencies (providing the same services at less cost), and c) resource release from scaling back or ceasing some services that may be of some benefit.
6. Evaluate investments and disinvestments in terms of costs and performance against criteria established in step 4 and make recommendations for a) funding growth areas with new resources and b) moving resources from 5b and 5c to 5a.
7. Validate results and reallocate resources.

In addition to the usual steps, public values were also fed in from repeated WTP surveys as one of the decision-making criteria. The whole process followed is outlined in the Figure.

**Figure.**
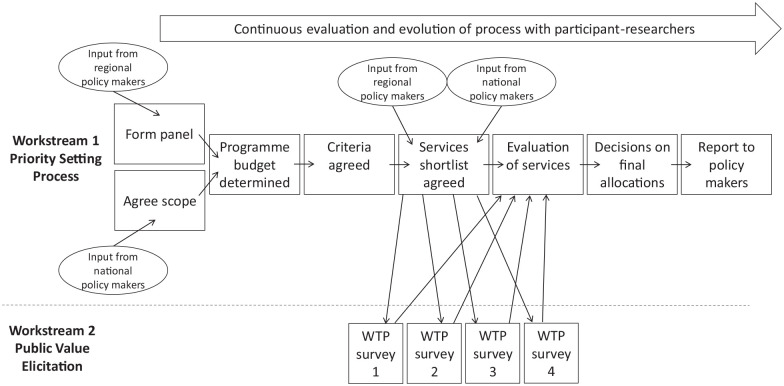
Overview of the program budgeting and marginal analysis process undertaken. WTP, willingness to pay.

### Scope of PBMA

While the PBMA was initiated by the research team, early engagement with the national policy makers was sought to ascertain that the PBMA would be useful and supported. In order to remain impartial, and for the panel to feel free to express their honest opinions, it was established early in the design that the process should be independent of the key decision makers at NHS England and that the findings would be presented to NHS England decision makers for consideration. It was agreed that the process should be cost-neutral (i.e., that no savings in the overall budget were being sought but that new investment was not available). The panel, at its first meeting, agreed the process should be undertaken at a whole country (England) level and broad in scope, including public money spent on oral health outside of the NHS. In practice, this meant that local government (local authority) activity in oral health promotion and epidemiology would be included.

### Panel Composition and Recruitment

The PBMA was designed to provide recommendations to national-level decision makers in NHS England dentistry, but the findings would be implemented by regional dental commissioners (NHS managers with responsibility for contracting for dental services) and their clinical advisors, termed local dental network (LDN) chairs. A setup workshop was therefore held with national decision makers, all regional dental commissioners and LDN chairs to determine the panel composition. The final agreed-on composition was 3 LDN chairs, 2 dental commissioners, 2 dental public health consultants, 2 patient/public members, 1 health economist, and 1 dental academic (who acted as facilitator). An email invitation was sent to LDN chairs, commissioners, and dental public health consultants with a participant information sheet. Those responding positively were selected purposively to ensure a diversity of individuals from different regions. Public representatives were approached through an existing patient and public involvement group at Newcastle University. All selected participants consented to their involvement as participant-researchers.

### Program Budget Definition

In order to determine the national NHS spend across different categories of dental care (see Table 1), all regional dental commissioners were emailed a template spreadsheet to complete, detailing the spend in 2015–2016. To capture non-NHS government spending (epidemiology and oral health promotion activities are funded by local government), dental public health consultants were emailed with a similar request. Mean spend per capita on different dental services was calculated for all regions with a response (9 of 16 NHS commissioning regions and 20 of 359 local government authorities), and total national spend was estimated by multiplying by total population. While the majority of spend falls into general dental services (primary care routine services), regional commissioners were unable to break this spend down due to the nature of contracts. Therefore, nationally published data from the dental claims processor (NHS Business Services Authority) were used to determine the number of items for each treatment type. This was then multiplied by the average fee paid for these treatments, and then a correction factor was applied as multiple items may be included in 1 fee payment. The panel reviewed these data and used them to inform further stages of the PBMA.

**Table 1.** Estimated Percentage Spend on Different Areas out of Total Dental Budget.

Service Type	Subtype	% Spend in 2015–2016 Financial Year
**General dental practice**	**Total**	**55**
	Examination	15
	Restorations	13
	Scale and polish	6
	Dentures	6
	Crown(s)	4
	Radiograph(s)	4
	Extractions	3
	Fluoride varnish	2
	Inlay(s)	<1
	Endodontics	<1
	Bridge(s)	<1
	Veneer(s)	<1
	Fissure sealants	<1
**Oral surgery**		16
**Orthodontics**		11
**Special care dentistry**		4
**Out of hours**		2
**Restorative dentistry**		2
**Pediatric dentistry**		1
**Sedation**		<1
**Small specialties (e.g., oral medicine/radiology)**		<1
**Unspecified** ^a^	**Total**	**9**
	Community dental services	4
	Hospital dental services	4
	Referral management systems	<1
**Local authority**	**Total**	**<1**
	Epidemiology	<1
	Health promotion	<1

a For these aspects, commissioners had contracts with a provider but did not specify how the contract should be split between specialty areas.

### Creation of Criteria and Scoring System

The panel had an open discussion about what criteria any program offered in the NHS dental service should fulfill. Ideas were refined in the discussion into a series of main criteria headings. The final list was agreed on by voting within the panel. The research team then developed a series of subcriteria, based on review of literature and in further discussion with the panel and within the academic team. The panel weighted each criterion and subcriterion on a percentage basis using anonymous electronic voting. First, the 10 panel members were asked individually to assign 100 points across the main criteria. The mean number of points for each criterion was taken as the final percentage weighting. Then each main criterion was taken in turn, and the panel members were asked individually to assign 100 points between the subcriteria for that main criterion. Again, the mean number of points was taken to give the percentage weighting for each subcriterion.

The research team then created a scoring system for each subcriterion. This was based on a previous PBMA scoring system (Carter 2000), which used a −2 to +2 scale for each subcriterion. Where possible, wording for the scoring system was adapted from the previous PBMA, but in many cases, the criteria were dental specific and so new scoring systems had to be created. The new criteria were discussed, amended, and agreed on by the panel.

### Selection of Program Components for Consideration

A series of program components for consideration for new investment, considerations for disinvestment, and program components that could be delivered more efficiently was agreed on in 2 stages. First, a long list was created from individual interviews with dental lead commissioners (methods reported elsewhere; Vernazza et al. 2019); a meeting with the national dental team, regional commissioners, and their clinical advisers; and in PBMA panel discussions. The second stage involved the panel voting on the long-listed program components to create a shortlist for onward consideration in the “marginal analysis” part of the PBMA process.

### Evidence Gathering and Scoring

The research team took each of the program components shortlisted by the panel and gathered evidence to support scoring. Most evidence was found using publicly available sources (such as published NHS data from NHS Digital and the NHS Business Service Authority) and from a systematic search of the academic literature. Three criteria required external sources of opinion to provide evidence. First, a survey was sent to all members of Parliament (MPs) by email to ascertain the political acceptability of each program component. For each program component, MPs rated the component on a 5-point scale (which corresponded to the −2 to +2 scale used for the criteria) and a median was taken. Only 16 of 650 MPs responded. Second, a public and patient involvement group at Newcastle University was used to give acceptability scores. Again, each member of the group (*n* = 7) was asked to rate each program component on a 5-point scale and a median was taken. Finally, a survey of a representative sample of the English public was undertaken to elicit preferences for each program component using contingent valuation methodology to determine WTP values (workstream 2 in the Figure). Respondents were presented with detailed information about each program component, and their value of having these program components provided by the NHS was determined by asking them to state their maximum WTP in increased taxation per annum for each option and aggregating these across the survey sample. The survey was undertaken in several waves to allow programs to be fed into the survey at various time points as the panel selected them and also to ensure that respondents were not overburdened with too many valuation tasks at any one time. In total, 339 participants valued all program components. The full methodology for the survey is described elsewhere (Vernazza et al. 2018).

All of the evidence was then presented to the panel, and following discussion, individuals scored each program component on each subcriterion in turn using anonymous electronic scoring. A median of the scores was taken.

### Final Reallocation Decisions

Panel members were individually emailed a spreadsheet-based visual tool that illustrated the program components under consideration, their overall scores, and their overall costs. Within the tool, individuals could select or deselect program components for inclusion in the NHS dental budget, and a sliding bar illustrated the overall resource available for any given combination of program components. Efficiency program components (components where the service would still be available but could be delivered in a different way, in the same volume but at a lower cost) were assumed to be implemented, and the resource saved from implementing these program components was added to the available budget. Individuals were asked to submit a set of recommendations that did not result in an overspend.

Responses from the panel members were then combined, and the total number of votes for each component was counted. Program components with 6 or more votes for either investment or disinvestment were agreed on as recommendations.

## Results

The program budget is shown in Table 1, detailing the percentage split of the total spend across England by different dental specialty areas. The estimated split of spend within the General Dental Service (primary care dental practices) is also shown.

The criteria developed by the panel and their weightings are shown in Table 2. The detailed definitions and the scoring system are available in the Appendix.

**Table 2.** Criteria for Program Budgeting and Marginal Analysis with Weightings.

Criterion	Weight, %	Subcriterion	Weight within Main Criterion, %	Overall Subcriterion Weight^a^
**Benefit**	22.8	Evidence base	26	5.9
		Size of problem	15.4	3.5
		Volume service treats	14.9	3.4
		Quality of life improvement	19.9	4.5
		Longevity of benefit	14	3.2
		Societal benefit (WTP survey)	9.9	2.3
**Cost-benefit**	12.8	Total cost less societal benefit	100	12.8
**Preventative**	13.9	Prevention level	54	7.5
		Evidence base	46	6.4
**Inequalities**	11.8	Effect on vertical inequality	45.8	5.4
		Effect on horizontal inequality	54.2	6.4
**Safe/acceptable**	9.1	Risk of untoward complication	26.8	2.4
		Longevity of side effects	27.6	2.5
		Pain associated with procedure	19.2	1.8
		Acceptable to patients	26.3	2.4
**Cost**	8.2	“Units of dental activity”^b^ generated per minute	18.4	1.5
		Cost per quality of life improvement	47.4	3.9
		Total cost	34.2	2.8
**Workforce**	7.2	Need to retrain workforce	55	4
		Uses dental care professionals	45	3.2
**Patient responsibility**	5.8	Onus on patient to care for oral health	100	5.8
**Innovation**	2.9	Current usage	100	2.9
**Politically acceptable**	2.8	MPs would find acceptable	100	2.8
**Aesthetics**	2.7	Intended to affect aesthetics	40	1.1
		Evidence base for aesthetic effect	60	1.6

MP, member of Parliament; WTP, willingness to pay.

a The overall subcriterion weight was determined by multiplying the main criterion by the subcriterion weight (e.g., the overall weight given to the benefit criterion was 22.8%). This was split into 6 subcriteria, each with its own relative weight. As the evidence base was given a weight of 26%, but this is of the total 22.8% weight given to the benefit criterion, the overall subcriterion weight is (22.8 * 0.26) = 5.9.

b Primary care dental contracts in England are based around the provider producing a specified number of “units of dental activity” based on a banded weighted tariff per course of treatment.

The program components/services considered as part of the PBMA process are as follows:

### New Investments

- Dental public health (DPH) input into local authority contracts: This would increase the dental public health support in local authorities so that they could ensure relevant oral health clauses were included in contracts for their services (such as contracts for school meals or for care homes).
- Expanding dental services in care homes: This would expand the offer of dental treatment, including simple restorations (using the atraumatic restorative technique), within the care home setting using portable equipment.
- Cognitive behavioral therapy (CBT): This would offer 6 to 10 CBT sessions with a trained therapist to develop coping mechanisms to enable dentally anxious patients to have dental treatment.
- Link to NHS helpline: This service would offer a direct booking with dental practices by the national NHS helpline service when a caller requires a dental appointment.
- Dental care for the homeless: This would expand drop-in dental care to homeless people via mobile outreach centers and fixed rehabilitation centers.
- New dental practice places in oversubscribed areas: This would offer more places with NHS dental practices in areas that experience excess demand.
- Preventative advice in practice: This would offer patients a 10-min prevention session with an oral health professional. This would include new remuneration for the practice for this activity.

### Efficiency Areas (Same Output but at Less Cost)

- Oral surgery in general dental practices: This intervention would make a range of treatments that need to be carried out by a specialist oral surgeon available in a local dental practice, rather at a referral center (typically in a hospital).

### Disinvestments

- Molar root canal: This would remove all root canal treatments on nonvital molar teeth to all adults (this would mean that only extraction would be offered on the NHS).
- Orthognathic surgery: This would stop orthognathic surgery, currently carried out for those with “great” or “very great need” on the Index of Orthognathic Functional Treatment Need (IOFTN) scale (Ireland et al. 2014). Note that orthognathic surgery carried out for patients with cleft lip and/or palate was not considered as part of this process and so this would continue.
- Moderate-need orthodontics (Index of Orthodontic Treatment Need [IOTN] 3): This would remove orthodontic provision for those younger than 16 years with IOTN (Brook and Shaw 1989) dental health component scores of 3 and with aesthetic component scores of 6 or above (those below this threshold are already not eligible for NHS treatment).
- Moderate- and severe-need orthodontics: This would remove orthodontic provision for those younger than 16 years with IOTN dental health component scores of 4 and those with dental health component scores of 3 and with aesthetic component scores of 6 or above (i.e., only those with IOTN scores of 5 would be eligible).
- Adult orthodontics: While adult orthodontics is in theory not available on the NHS, there is variable availability across the country, and this would completely stop all adult orthodontics in primary care (secondary care settings carry out orthodontics for patients with dentofacial deformities beyond the age of 16 y, and this was not considered in this program component).
- Routine scale and polish: This would stop scaling and polishing for those patients with periodontal disease, which is often carried out for those without periodontal disease, usually at the same interval as a dental checkup.
- Out-of-hours dental pain service: This would remove the current service at evenings and weekends (usually a telephone triage and, if required, a dental appointment) to patients in pain (the service would continue for those with trauma, bleeding, and swelling).

The scores for each subcriterion, as well as the overall weighted scores for each program component, are shown in Table 3. A worked example of the scoring for 1 program component (out-of-hours dental pain service) is available in the Appendix.

**Table 3.** Scores for Each Program Component by Each Criterion.

Characteristic	Benefit	Cost	Cost-Benefit	Preventive	Health Inequalities	Safe/ Acceptable	Workforce	Politically Acceptable	Increases Patient Responsibility	Innovative	Aesthetic Results	Final Weighted Score
**Out-of-hours pain**	0.73	1.29	2.00	0.00	1.54	0.94	0.65	1.00	0.00	−2.00	0.00	0.81
**Helpline link**	0.42	−0.26	1.00	0.00	1.54	2.00	2.00	1.00	0.00	−0.50	0.00	0.72
**Extra DPH input**	0.68	−0.26	−1.00	1.31	1.54	2.00	−1.00	1.00	1.50	−1.00	0.00	0.57
**Care homes**	−0.57	0.82	2.00	0.46	1.31	1.54	0.00	1.00	0.00	−1.00	0.40	0.56
**Preventive sessions**	0.89	−0.10	−2.00	1.54	−0.77	2.00	0.35	2.00	2.00	−1.00	1.00	0.44
**New practices**	−0.15	−0.61	1.00	0.46	−0.46	2.00	−1.00	2.00	1.00	−2.00	0.40	0.23
**Moderate + severe ortho**	0.32	−0.82	1.00	0.00	0.00	−0.37	0.65	1.00	0.00	−2.00	1.40	0.16
**CBT**	0.61	−1.16	−2.00	0.00	−0.27	1.90	1.45	1.00	1.00	−1.00	0.00	0.09
**Jaw surgery**	−0.66	−0.61	2.00	0.00	0.27	−1.21	0.65	1.00	0.00	−2.00	1.40	0.03
**Homeless care**	−0.66	−0.82	−2.00	0.46	1.73	1.07	0.10	2.00	0.50	−1.00	1.00	−0.02
**Routine scaling**	0.50	−1.66	−1.00	−0.73	0.04	1.54	1.55	0.00	0.00	−2.00	0.40	−0.04
**Molar endodontics**	0.18	−1.29	1.00	−0.92	−0.54	0.20	0.65	1.00	0.00	−2.00	0.00	−0.09
**Adult orthodontics**	−0.01	0.21	−1.00	0.00	−0.77	−0.37	0.65	1.00	0.00	−2.00	1.40	−0.18
**Moderate orthodontics**	0.09	−0.13	−1.00	0.00	−0.77	−0.63	0.65	1.00	0.00	−2.00	1.40	−0.21

CBT, cognitive behavioral therapy; DPH, dental public health.

Despite repeated reminders, 1 panel member did not return their final recommendations and so 9 individuals contributed to this stage. Table 4 shows the number of votes each program component received alongside its cost and score, as well as the final decision. The final allocations (changes to program components with 6 votes or more enacted) left an unspent budget of £134 million.

**Table 4.** Number of Votes and Final Recommendation for Each Program.

Service	Currently Provided	Estimated Cost (£ Millions)	Score (−2 to +2 Scale)	No. of Panel Investing (*n* = 9)	Recommend for Funding
**Moderate-need orthodontics**	Yes	44	−0.21	3	Disinvest
**Adult orthodontics**	Yes	11	−0.18	3	Disinvest
**Routine scaling**	Yes	174	−0.04	2	Disinvest
**Molar endodontics**	Yes	190	−0.09	6	Continue
**Orthognathic surgery**	Yes	20	0.03	7	Continue
**Moderate- + severe-need orthodontics**	Yes	99	0.16	7	Continue
**Out-of-hours pain**	Yes	54	0.81	7	Continue
**Dental care for the homeless**		190	−0.02	5	No investment
**Cognitive behavioral therapy**		824	0.09	0	No investment
**Preventative sessions**		659	0.44	0	No investment
**New practice places**		135	0.23	7	Invest
**Care home work**		13	0.56	8	Invest
**Dental public health input**		5	0.57	7	Invest
**National Health Service helpline link**		2	0.72	6	Invest

## Discussion

This is the first article to describe a resource allocation process using the PBMA framework in a national dental context. Although the recommendations of the process are specific to the country and the timeframe that the process was undertaken in, this article establishes a framework and describes steps that can be undertaken elsewhere. As noted in the Introduction, there are different measures of success of a PBMA (Tsourapas and Frew 2011), and this study could be seen to be successful in increasing understanding and awareness of the resource allocation issues in the given area. It is too early yet to ascertain success on other criteria of enacting of recommendations and embedding of PBMA within the organization, but work is ongoing to monitor this.

Although work is ongoing to assess the uptake of recommendations, initial discussions with policy makers have highlighted concerns over whether the recommendations can be operationalized within the confines of legislation and dental contracts. Other concerns have centered on problems specific to the NHS context in England with a lack of clarity about whether these changes would be implemented at local or national levels. These concerns are not unique to this project, with a review of PBMA studies finding that the context was the most frequently cited barrier to ongoing use and uptake of recommendations (Mitton and Donaldson 2001).

The results generally favor access-related program components (e.g., more general dental places, care home dentistry but not care for the homeless) while disinvesting from more appearance-based program components (e.g., orthodontics). Early methodological testing of the WTP survey (Carr et al. 2021) showed there was not a high degree of discrimination between WTP values, which may indicate a lack of preference between programs. Later values collected showed higher values for appearance-based program components, with lower values for some of the access-related program components (i.e., the opposite to the final recommendations). In constructing the criteria and weighting them, it can be seen that the panel gave a relatively small proportion of the overall score to the public opinion via the survey (the societal benefit subcriteria). Whether this is correct or not is a matter for debate and further research, and including qualitative interviews with the stakeholders as well as focus groups with respondents to the WTP survey is planned to investigate this question further. It is important to note that the survey was 1 of 2 avenues to include the public in the PBMA exercise as the public were also represented on the panel. The questions of who should be asked for views and how to do this in a representative but informed manner remain important for further investigation.

Another interesting finding was that while many panel members expressed a strong desire to invest in the preventive program component, this was impossible, because even if all other program components had been disinvested from, there would not have been enough money released to invest in this single program component given the parameters set by the program component description. This was also true of the CBT program component, although fewer panel members expressed concerns about not being able to invest in this program component. This also indicates another contribution of PBMA, in identifying areas of care that would be prioritized if budgets were expanded. On the other hand, the panel was also concerned that there was a significant amount left over unspent. Here, consideration must be given to the divisibility of the program components, in that program components were considered nondivisible (i.e., funding part of a program component or introducing a program component for limited numbers of people was not allowed). This was because the evidence had been gathered and opinions sought from groups (including the WTP survey) on the basis of a whole program component, and if only part of program component was funded, this would have potentially changed its score. One of the criticisms of quality-adjusted life year threshold-based approaches often used by national health technology assessment agencies for deciding on allocations is that this assumes complete divisibility of a program components, often where this would not be possible. It seems that this issue is worthy of further consideration in future PBMAs. However, in this example, decision makers may pragmatically wish to partially invest the leftover resource in program components that scored well or were nearly funded. In addition, this PBMA reflected the current situation, but objectives will change over time, and if recommendations are enacted, the current allocation and outputs will also change. This implies that the PBMA process should be iterative and perhaps be viewed as an ongoing process where changes are enacted and evaluated, and then further changes are made. However, it is not clear how frequently such an exercise should be repeated given the resources required to run it.

The limitations of the study are mainly criticisms of the PBMA approach more generally. There have been concerns expressed about the makeup of PBMA panels (Marsh et al. 2018), with the potential for different stakeholders to influence the outcomes in different ways, given the subjective nature of some of the decisions. In particular, the final step of making recommendations only involves consideration of the scores rather than these being used directly as part of a decision rule. This is necessary because, at this stage, the overall costs and budget must be considered at the same time as the scores, making a subjective consideration necessary. In this case, the problem of using the subjective opinions of a small panel was partially mitigated by involving wider groups such as the workshop of commissioners and LDN chairs, the public survey and opinions from MPs, and patient groups. Unfortunately, the survey of MPs only generated a very limited response, perhaps reflecting the importance of oral health in their overall portfolio of interests. It may be that future PBMAs’ political acceptability should be scored in different ways, drawing on a group more closely interested in oral health. In addition, panel members were reminded that they were representing groups and were encouraged to discuss points with colleagues between panel meetings. More widely, the PBMA process has been defended on the basis that the discussions involve all stakeholders rather than isolated decision makers, as is often the case currently, and that the decisions are therefore made more transparent (Hall, Mitton, et al. 2018). Despite the challenges of PBMA, it can offer a better alternative to repeated historical allocation or “decibel” approaches and can provide an indication of the direction of future travel even if results are not immediately implementable.

## Conclusion

This study illustrates a PBMA completed successfully (in terms of raised awareness) at a national level for dentistry. Implementation of the recommendations will depend on the ability of policy makers to operationalize the findings. The study shows several practical aspects that could be adopted for future PBMAs, including the criteria, scoring system, and conduct of the process.

## Author Contributions

C.R. Vernazza, contributed to conception, design, data acquisition, analysis, and interpretation, drafted and critically revised the manuscript; K. Carr, R.D. Holmes, contributed to design, data acquisition, analysis, and interpretation, critically revised the manuscript; J. Wildman, J. Gray, C. Exley, contributed to design, data analysis, and interpretation, critically revised the manuscript; R.A. Smith, contributed to design and data interpretation, critically revised the manuscript; C. Donaldson, contributed to conception, design, data analysis, and interpretation, critically revised the manuscript. All authors gave final approval and agree to be accountable for all aspects of the work.

## Supplemental Material

sj-docx-1-jct-10.1177_23800844211056241 – Supplemental material for Resource Allocation in a National Dental Service Using Program Budgeting Marginal Analysis Click here for additional data file. Supplemental material, sj-docx-1-jct-10.1177_23800844211056241 for Resource Allocation in a National Dental Service Using Program Budgeting Marginal Analysis by C.R. Vernazza, K. Carr, R.D. Holmes, J. Wildman, J. Gray, C. Exley, R.A. Smith and C. Donaldson in JDR Clinical & Translational Research
