# Supplementary material for: Resource Allocation in a National Dental Service Using Program Budgeting Marginal Analysis
Source: JDR Clin Trans Res. 2021 Nov 29;8(1):56–65. doi: 10.1177/23800844211056241 (PMC9772892; doi:10.1177/23800844211056241)
Supplement: sj-docx-1-jct-10.1177_23800844211056241 – Supplemental material for Resource Allocation in a National Dental Service Using Program Budgeting Marginal Analysis [file sj-docx-1-jct-10.1177_23800844211056241.docx]

**Resource allocation in a national dental service using Programme Budgeting Marginal Analysis**

**Supplementary material**

1. **Full criteria and scoring system**

**Benefit**

| **Domain** | **Weight** | **Criteria** | **Definition** | **Criteria Weight** | **Rating Scale** | | | | |
| --- | --- | --- | --- | --- | --- | --- | --- | --- | --- |
|  |  |  |  |  | **-2** | **-1** | **0** | **1** | **2** |
| Benefit | **Average:**  **22.8%** | Effectiveness & efficacy | 1) evidence base for intervention | 26% | No evidence | Effect due to chance | Inconclusive evidence | Effect is weakly linked to intervention | Effect is strongly linked to intervention |
|  | LDN chair:  20% |  |  |  |  |  |  |  |  |
|  |  | Health Impact | 2) Size of problem (where can the biggest difference be made?) | 15.4% | Less than 200,000 have this condition | Between 200,001 and 500,000 have this condition | Unknown or inconclusive | Between 500,001 and 1 million have this condition | Over 1 million have this condition |
|  | DPH consultant:  22.5% |  |  |  |  |  |  |  |  |
|  | PPI:  37.1% |  |  |  |  |  |  |  |  |
|  |  |  | 3) volume treated | 14.9% | Under 150,000 would be treated | Between 150,001 and 500,000 would be treated | Unknown | Between 500,000 and 1 million would be treated | Over 1 million would be treated |
|  | Commissioner:  13.5% |  |  |  |  |  |  |  |  |
|  | RAINDROP staff:  22% |  |  |  |  |  |  |  |  |
|  |  |  | 4) size of benefit (proportional QoL improvement) | 19.9% | Over 5% decrease in QoL | Between 0 and 5% decrease in QoL | Average QoL score would be unchanged or unknown | Between 0 and 5% QoL improvement | Over 5% QoL improvement |
|  |  |  | 5) Longevity of benefit | 14% | Intervention may require repetition once/ < 6m or ongoing maintenance | Intervention may require repetition 6m & 2 yrs or frequent maintenance | Unknown | Intervention may require repetition 2yrs + or infrequent maintenance | Intervention does not require repetition or maintenance |
|  |  |  | 6) societal benefit | 9.9% | In quarter of most protested interventions | Next to bottom quarter of most protested interventions | Unknown | Next to top quarter of least protested interventions | In quarter of least protested interventions |

**Cost**

| **Domain** | **Weight** | **Criteria** | **Definition** | **Criteria Weight** | **Rating Scale** | | | | |
| --- | --- | --- | --- | --- | --- | --- | --- | --- | --- |
|  |  |  |  |  | **-2** | **-1** | **0** | **1** | **2** |
| Cost | **Average:**  **8.2%** | Time | UDA per minute | 18.4% | 0.11 or under gained per minute | Between 0.11 and 0.24 gained per minute | Not applicable (non-GDS intervention) | Between 0.25 and 0.49 gained per minute | 0.5 or more gained per minute |
|  | LDN Chairs:  5% |  |  |  |  |  |  |  |  |
|  | DPH consultants:  13.5% | Efficiency | Cost per QoL % improvement | 47.4% | No/less than 1% QoL improvement but costs incurred | 1% QoL improvement for £15+ | No QoL improvement, no additional costs | 1% QoL improvement for £7.50-14.99 | 1% QoL improvement for £0-7.50 |
|  | PPI:  5.6% |  |  |  |  |  |  |  |  |
|  | Commissioner:  12.5% |  |  |  |  |  |  |  |  |
|  | RAINDROP staff:  16% |  |  |  |  |  |  |  |  |
|  |  | Monetary cost | Sum cost of providing treatment | 34.2% | Most expensive quarter of ranked interventions | Next to bottom quarter of ranked interventions | Unknown | Next to top quarter of ranked interventions | Least quarter of ranked interventions |

**Cost-benefit**

| **Domain** | **Weight** | **Criteria** | **Definition** | **Criteria Weight** | **Rating Scale** | | | | |
| --- | --- | --- | --- | --- | --- | --- | --- | --- | --- |
|  |  |  |  |  | **-2** | **-1** | **0** | **1** | **2** |
| Cost-benefit | **Average:**  **12.8%** | Cost-benefit | Net social benefit (WTP-Cost) | 100% | Bottom quarter of ranked interventions | Next to bottom quarter of ranked interventions | Unknown | Next to top quarter of ranked interventions | Top quarter of ranked interventions |
|  | LDN chair:  15% |  |  |  |  |  |  |  |  |
|  | DPH consultant:  13.5% |  |  |  |  |  |  |  |  |
|  | PPI:  6.9% |  |  |  |  |  |  |  |  |
|  | Commissioner:  12% |  |  |  |  |  |  |  |  |
|  | RAINDROP staff:  18% |  |  |  |  |  |  |  |  |

**Preventive**

| **Domain** | **Weight** | **Criteria** | **Definition** | **Criteria Weight** | **-2** | **-1** | **0** | **1** | **2** |
| --- | --- | --- | --- | --- | --- | --- | --- | --- | --- |
| Preventive | **Average:**  **13.9%** | Level of prevention for oral disease | What preventive level is the intervention? | 54% | Increases disease | Condition is preventable but intervention not preventive | Tertiary prevention or disease is not preventable | Secondary prevention | Primary prevention |
|  | LDN chair:  16.7% |  |  |  |  |  |  |  |  |
|  | DPH consultant:  20% |  |  |  |  |  |  |  |  |
|  |  | Evidence of preventive effect | What level of evidence is there for the preventive effect | 46% | No evidence | Effect due to chance | Inconclusive evidence or disease is not preventable | Effect is weakly linked to intervention | Effect is strongly linked to intervention |
|  | PPI:  6.1% |  |  |  |  |  |  |  |  |
|  | Commissioner:  12.5% |  |  |  |  |  |  |  |  |
|  | RAINDROP staff:  12% |  |  |  |  |  |  |  |  |

**Decreases health inequalities**

| **Domain** | **Weight** | **Criteria** | **Definition** | **Criteria Weight** | **Rating Scale** | | | | |
| --- | --- | --- | --- | --- | --- | --- | --- | --- | --- |
|  |  |  |  |  | **-2** | **-1** | **0** | **1** | **2** |
| **Health inequalities** | **Average:**  **11.8%** | Equity & equality | 1) Impact on the health status of recognised groups where there is a known health status gap (vertical) | 45.8% | Intervention specifically targets those not at risk | Intervention is more likely to affect those not at risk | No discrimination between groups | Intervention is more likely to impact at risk groups | Intervention specifically targets at risk groups |
|  | LDN chair:  9.7% |  |  |  |  |  |  |  |  |
|  | DPH consultant:  20% |  |  |  |  |  |  |  |  |
|  |  |  | 2) Equal access for equal need (horizontal) | 54.2% | Intervention discriminates between those who have equal need | Evidence of self-selection for intervention | Unknown | Evidence of intervention being utilised by those with equal need despite discriminatory provision | Intervention provided and utilised for all who have equal need |
|  | PPI:  5% |  |  |  |  |  |  |  |  |
|  | Commissioner:  14% |  |  |  |  |  |  |  |  |
|  | RAINDROP staff:  12.5% |  |  |  |  |  |  |  |  |

**Safe/Acceptable**

| **Domain** | **Weight** | **Criteria** | **Definition** | **Criteria Weight** | **-2** | **-1** | **0** | **1** | **2** |
| --- | --- | --- | --- | --- | --- | --- | --- | --- | --- |
| Safe/Acceptable | **Average:**  **9.1%** | Safe | What is the risk of untoward complications? | 26.8% | Risk >10% of untoward complications | Risk 6-10% of untoward complications | 1-5% risk of untoward complications | 0-1% risk of untoward complication | No untoward complications at all |
|  | LDN chairs:  15% |  |  |  |  |  |  |  |  |
|  | DPH consultants:  5% |  |  |  |  |  |  |  |  |
|  |  |  | What is the longevity of side effects? | 27.6% | Longer than 6 months | 1 month to 6 months | 1 week to 1 month | 2 days to 1 week | Less than 2 days or no side effect |
|  | PPI:  4.8% |  |  |  |  |  |  |  |  |
|  | Commissioner:  8% |  |  |  |  |  |  |  |  |
|  |  | Acceptable | Does carrying out the procedure cause pain/discomfort? | 19.2% | Severe discomfort that would require sedation/GA in most cases | Discomfort/pain likely even with LA | Procedure requires LA to be completed | Mild discomfort during procedure | No pain/ discomfort |
|  | RAINDROP staff:  10% |  |  |  |  |  |  |  |  |
|  |  |  | Acceptable to patients | 26.3% | Ask patient panel to rank on 1-5 scale of Fully acceptable to Unacceptable in terms of procedure itself, after-effects and long term consequences | | | | |

**Best use of workforce**

| **Domain** | **Weight** | **Criteria** | **Definition** | **Criteria Weight** | **-2** | **-1** | **0** | **1** | **2** |
| --- | --- | --- | --- | --- | --- | --- | --- | --- | --- |
| Work force | **Average:**  **7.2%** | Utilises existing workforce | Likelihood of needing to re-train/re-orientate existing workforce | 55% | Proposal deliverable only by majorly changing workforce | Proposal deliverable only by slight changes to workforce | Proposal deliverable by current workforce with major training (more than one day course) | Proposal deliverable by current workforce with minor training (e.g. one day course) | Proposal deliverable by current workforce with no change to working patterns |
|  | LDN chairs:  4.8% |  |  |  |  |  |  |  |  |
|  | DPH consultant:  3% |  |  |  |  |  |  |  |  |
|  | PPI:  12.1% |  |  |  |  |  |  |  |  |
|  | Commissioner:  12.5% |  |  |  |  |  |  |  |  |
|  | RAINDROP staff:  2% |  |  |  |  |  |  |  |  |
|  |  | Uses DCPs | Does the proposal increase the skill-mix use? | 45% | Proposal designed to decrease use of skill mix | Proposal could not be delivered by non-dentists | Proposal could be delivered by non-dentists but not likely | Proposal likely to be delivered by non-dentists | Proposal directly designed to be delivered by non-dentists |

**Politically acceptable**

| **Domain** | **Weight** | **Criteria** | **Definition** | **Criteria Weight** | **-2** | **-1** | **0** | **1** | **2** |
| --- | --- | --- | --- | --- | --- | --- | --- | --- | --- |
| Politically acceptable | **Average:**  **2.8%** | Politically acceptable | Would change (i.e. disinvestment or investment) be likely to be enacted if presented to minister | 100% | Ask MPs to rank on 1-5 scale from Unacceptable-Acceptable-Positive | | | | |
|  | LDN chair:  2% |  |  |  |  |  |  |  |  |
|  | DPH consultant:  2% |  |  |  |  |  |  |  |  |
|  | PPI:  4% |  |  |  |  |  |  |  |  |
|  | Commissioner:  3% |  |  |  |  |  |  |  |  |
|  | RAINDROP staff:  4% |  |  |  |  |  |  |  |  |

**Increases patient responsibility**

| **Domain** | **Weight** | **Criteria** | **Definition** | **Criteria Weight** | **-2** | **-1** | **0** | **1** | **2** |
| --- | --- | --- | --- | --- | --- | --- | --- | --- | --- |
| Increases patient responsibility | **Average:**  **5.8%** | Increases patient responsibility | Does the intervention place an onus on the patient to take control of their oral health | 100% | Intervention will directly decrease patient responsibility | Intervention will indirectly decrease patient responsibility | Intervention has no effect on patient responsibility | Intervention will indirectly increase patient responsibility | Intervention directly related to increasing patient responsibility |
|  | LDN chair:  9.2% |  |  |  |  |  |  |  |  |
|  | DPH consultant:  3% |  |  |  |  |  |  |  |  |
|  | PPI:  5% |  |  |  |  |  |  |  |  |
|  | Commissioner:  7% |  |  |  |  |  |  |  |  |
|  | RAINDROP staff:  0% |  |  |  |  |  |  |  |  |

**Innovative**

| **Domain** | **Weight** | **Criteria** | **Definition** | **Criteria Weight** | **-2** | **-1** | **0** | **1** | **2** |
| --- | --- | --- | --- | --- | --- | --- | --- | --- | --- |
| Innovative | **Average:**  **2.9%** | Current usage | How widespread is current usage? | 100% | Intervention already used routinely in England | Intervention already used routinely in some practices or not in England | Intervention used outside of England in routine practice | Intervention used outside of England or in research settings but only occasionally in routine practice | Intervention not used anywhere in clinical practice (may be used in research setting either in UK or abroad) |
|  | LDN chair:  1.8% |  |  |  |  |  |  |  |  |
|  | DPH consultant:  2% |  |  |  |  |  |  |  |  |
|  | PPI:  5.4% |  |  |  |  |  |  |  |  |
|  | Commissioner:  3.5% |  |  |  |  |  |  |  |  |
|  | RAINDROP staff:  2% |  |  |  |  |  |  |  |  |

**Aesthetic results**

| **Domain** | **Weight** | **Criteria** | **Definition** | **Criteria Weight** | **-2** | **-1** | **0** | **1** | **2** |
| --- | --- | --- | --- | --- | --- | --- | --- | --- | --- |
| Aesthetic results | **Average:**  **2.7%** | Aesthetic intent | Is the intervention intended to affect aesthetics? | 40% | Intervention will directly negatively affect appearance of teeth/face | Intervention may indirectly affect appearance of teeth/face | Intervention does not have effect on appearance of teeth/face | Intervention is indirectly related to improving appearance of teeth/face | Intervention is directly related to improving appearance of teeth/face |
|  | LDN chair:  0.8% |  |  |  |  |  |  |  |  |
|  | DPH consultant:  1.5% |  |  |  |  |  |  |  |  |
|  | PPI:  7.9% |  |  |  |  |  |  |  |  |
|  | Commissioner:  3% |  |  |  |  |  |  |  |  |
|  |  | Evidence for aesthetic effect | Strong evidence (Systematic review or good RCT) intervention is likely to negatively affect aesthetics | 60% | Strong evidence of negative aesthetic effect | Weak evidence of negative aesthetic effect | Intervention unlikely to affect | Weak evidence (poor RCT or observational study) intervention is likely to improve aesthetics | Strong evidence (Systematic review or good RCT) intervention is likely to improve aesthetics |
|  | RAINDROP staff:  0% |  |  |  |  |  |  |  |  |

1. **Worked example of scoring for out of hours dental pain service**

| **Domain** | **Weight** | **Criteria** | **Definition** | **Weight** | **Scoring** | | | | | |
| --- | --- | --- | --- | --- | --- | --- | --- | --- | --- | --- |
|  |  |  |  |  | **Source** | **Notes** | **Score** | **Weighted score (Column 5 x Column 8)** | **Total score for main criterion** | **Weighted score for main criterion (Column 9 x Column 2)** |
| Benefit | 22.8% | Effectiveness & efficacy | 1) evidence base for intervention | 26% | Anderson R, Thomas DW, Phillips CJ (2005) The effectiveness of out-of-hours dental services: I. pain relief and oral health outcome. British Dental Journal 198, 91. | Only one cohort study found | -1 | -0.26 | 0.73 | 0.17 |
|  |  | Health Impact | 2) Size of problem (where can the biggest difference be made?) | 15.4% |  | Demand = Recent figures indicate 9.3% of all dental treatments are considered ‘urgent’ (bad enough to want immediate care but not bad enough to go to A&E). This is approximately 3.7 million dental treatments a year. Urgent care can also take place during normal working hours, but it is likely most of these treatments were done by dentists working out of hours.  Need will be larger than demand so > 1 million | 2 | 0.308 |  |  |
|  |  |  | 3) volume treated | 14.9% |  | As above | 2 | 0.298 |  |  |
|  |  |  | 4) size of benefit (proportional QoL improvement) | 19.9% | Anderson R, Thomas DW, Phillips CJ (2005) The effectiveness of out-of-hours dental services: I. pain relief and oral health outcome. British Dental Journal 198, 91. | 26% increase in Dental QoL measure | 2 | 0.398 |  |  |
|  |  |  | 5) Longevity of benefit | 14% |  | Difficult to quantify as most emergency attendances likely to need follow up treatment with normal service and evidence for repeat attenders | -1.5 | -0.21 |  |  |
|  |  |  | 6) societal benefit | 9.9% |  | Ranking of all programmes based on WTP values = 1 | 2 | 0.198 |  |  |
| Cost | Average:  8.2% | Time | UDA per minute | 18.4% |  | From programme budget work | 0 | 0 | 1.29 | 0.11 |
|  |  | Efficiency | Cost per QoL % improvement | 47.4% |  | Programme budget, split by service indicates costs £81,252,763.52 | 2 | 0.948 |  |  |
|  |  | Monetary cost | Sum cost of providing treatment | 34.2% |  | Ranking of all programmes | 1 | 0.342 |  |  |
| Cost-benefit | Average:  12.8% | Cost-benefit | Net social benefit (WTP-Cost) | 100% |  | Ranking of all programmes using above cost and Workstream 2 WTP values | 2 | 2 | 2 | 0.26 |
| Preventive | Average:  13.9% | Level of prevention for oral disease | What preventive level is the intervention? | 54% |  | If urgent care required – disease needs repairing but urgent care unlikely to do this | 0 | 0 | 0 | 0 |
|  |  | Evidence of preventive effect | What level of evidence is there for the preventive effect | 46% |  | Not preventive | 0 | 0 |  |  |
| **Health inequalities** | Average:  11.8% | Equity & equality | 1) Impact on the health status of recognised groups where there is a known health status gap (vertical) | 45.8% |  | Deprivation associated with dental pain | 1 | 0.458 | 1.54 | 0.18 |
|  |  |  | 2) Equal access for equal need (horizontal) | 54.2% |  | Available to all who require | 2 | 1.084 |  |  |
| Safe/Acceptable | Average:  9.1% | Safe | What is the risk of untoward complications? | 26.8% |  | Emergency provision itself does not have higher risks than usual treatment | 0.5 | 0.134 | 0.94 | 0.09 |
|  |  |  | What is the longevity of side effects? (if no SE = 2, Assume more than 50% have to have side effect at time point to be relevant) | 27.6% |  | No Side Effects | 1 | 0.276 |  |  |
|  |  | Acceptable | Does carrying out the procedure cause pain/discomfort? | 19.2% |  | Likely to require Local Anaethesia as part of service | 0 | 0 |  |  |
|  |  |  | Acceptable to patients | 26.3% |  |  | 2 | 0.526 |  |  |
| Work force | Average:  7.2% | Utilises existing workforce | Likelihood of needing to re-train/re-orientate existing workforce | 55% |  | Utilises existing workforce | 2 | 1.1 | 0.65 | 0.05 |
|  |  | Uses DCPs | Does the proposal increase the skill-mix use? | 45% |  | Relies on dentist as diagnosis required | -1 | -0.45 |  |  |
| Politically acceptable | Average:  2.8% | Politically acceptable | Would change (i.e. disinvestment or investment) be likely to be enacted if presented to minister | 100% |  | MPs survey - unacceptable | 1 | 1 | 1 | 0.03 |
| Increases patient responsibility | Average:  5.8% | Increases patient responsibility | Does the intervention place an onus on the patient to take control of their oral health | 100% |  | No | 0 | 0 | 0 | 0 |
| Innovative | Average:  2.9% | Current usage | How widespread is current usage? | 100% |  | Out of hours/emergency dentists are available nationally | -2 | -2 | -2 | -0.06 |
| Aesthetic results | Average:  2.7% | Aesthetic intent | Is the intervention intended to affect aesthetics? | 40% |  | No impact | 0 | 0 | 0 | 0 |
|  |  | Evidence for aesthetic effect | Strong evidence (Systematic review or good RCT) intervention is likely to negatively affect aesthetics | 60% |  | No impact | 0 | 0 |  |  |
| **TOTAL** |  |  |  |  |  |  |  |  |  | **0.81** |
